# Effects of Gradient Coil Noise and Gradient Coil Replacement on the Reproducibility of Resting State Networks

**DOI:** 10.3389/fnhum.2018.00148

**Published:** 2018-04-19

**Authors:** Epifanio Bagarinao, Erina Tsuzuki, Yukina Yoshida, Yohei Ozawa, Maki Kuzuya, Takashi Otani, Shuji Koyama, Haruo Isoda, Hirohisa Watanabe, Satoshi Maesawa, Shinji Naganawa, Gen Sobue

**Affiliations:** ^1^Brain and Mind Research Center, Nagoya University, Nagoya, Japan; ^2^Department of Radiological Technology, School of Health Sciences, Nagoya University, Nagoya, Japan; ^3^Department of Radiology, Nagoya University Graduate School of Medicine, Nagoya, Japan

**Keywords:** resting state networks, reproducibility, gradient coil noise, gradient coil replacement, resting state fMRI

## Abstract

The stability of the MRI scanner throughout a given study is critical in minimizing hardware-induced variability in the acquired imaging data set. However, MRI scanners do malfunction at times, which could generate image artifacts and would require the replacement of a major component such as its gradient coil. In this article, we examined the effect of low intensity, randomly occurring hardware-related noise due to a faulty gradient coil on brain morphometric measures derived from T1-weighted images and resting state networks (RSNs) constructed from resting state functional MRI. We also introduced a method to detect and minimize the effect of the noise associated with a faulty gradient coil. Finally, we assessed the reproducibility of these morphometric measures and RSNs before and after gradient coil replacement. Our results showed that gradient coil noise, even at relatively low intensities, could introduce a large number of voxels exhibiting spurious significant connectivity changes in several RSNs. However, censoring the affected volumes during the analysis could minimize, if not completely eliminate, these spurious connectivity changes and could lead to reproducible RSNs even after gradient coil replacement.

## Introduction

Resting state functional magnetic resonance imaging (fMRI) is an imaging technique that does not require participants to perform any task during the scan, making the approach attractive in investigating healthy brain functions as well as in examining changes occurring in the diseased brain. Resting state fMRI is commonly used to examine functional connectivity among spatially distributed brain regions that form the so-called resting state networks (RSNs; Greicius et al., 2003; Beckmann et al., 2005; Fox et al., 2005; Damoiseaux et al., 2006). In healthy population, resting state fMRI has been proven useful in investigating the very early development of these RSNs in infancy (Fransson et al., 2007; Smyser et al., 2010) and how these RSNs are transformed across the lifespan (Tomasi and Volkow, 2012; Betzel et al., 2014; Sala-Llonch et al., 2015). In clinical population, resting state fMRI has also been used to examine changes in functional connectivity within RSNs in chronic pain conditions (Baliki et al., 2008, 2014; Martucci et al., 2015; Li et al., 2016), Parkinson’s disease (Hacker et al., 2012; Szewczyk-Krolikowski et al., 2014; Yao et al., 2014), Alzheimer’s disease (Greicius et al., 2004; Dai et al., 2015), patients with brain tumor (Esposito et al., 2012; Maesawa et al., 2015), and other neuropsychological disorders (Greicius, 2008). Using resting state fMRI, RSNs have also been shown to be highly reproducible across participants (Beckmann et al., 2005; Chen et al., 2008) and within participants over multiple sessions (Damoiseaux et al., 2006; Chen et al., 2008; Choe et al., 2015), making resting state functional connectivity a promising biomarker for clinical applications (Shimony et al., 2009; Fox and Greicius, 2010; Lee et al., 2013).

The reproducibility of RSNs, however, could be affected by several factors. One of these factors is the hardware used to acquire the imaging dataset. For multisite studies, differences in MRI providers, magnet field strengths, software and coil architectures need to be considered. Even with the same MRI scanner, maintaining the scanner’s stability throughout a given imaging study is very important in minimizing hardware-related variability in the acquired imaging dataset. For cross-sectional studies, scanner stability ensures that the variability in any MRI-derived measures is mainly due to the population being studied. For longitudinal studies, scanner stability guarantees that changes observed over time are driven by disease progression, for example, and not by instrument-related factors. However, MRI scanners do malfunction at times, which could necessitate replacement of major components such as the scanner’s gradient coil. This major change in hardware could introduce unwanted variability into the imaging dataset. Faulty MRI scanner gradient coil could also generate artifacts in the acquired images, the degree of which could vary depending on the severity of the problem. Large artifacts are easier to detect but low intensity, randomly occurring artifacts are difficult to discover. Some low intensity gradient-coil-generated noise are barely noticeable, but have the potential to significantly affect the outcome of the analysis.

In this study, we examined the effects of low intensity gradient coil noise on resting state fMRI data on the reproducibility of RSNs and introduced a method to minimize these effects. We further examined whether RSNs constructed from resting state fMRI and values of regional gray matter (GM) volume estimated from T1-weighted images are reproducible using data acquired before and after gradient coil replacement. For this, we performed voxel-wise statistical comparisons of RSNs and GM between sessions to identify differences at the voxel level. To compare RSNs, we used group independent component analysis (ICA) and dual regression analysis (Filippini et al., 2009), which have been found effective and reliable in analyzing resting state fMRI data (Zuo et al., 2010). To assess differences in GM, we used voxel-based morphometry (VBM; Ashburner and Friston, 2000), a commonly used approach for analyzing T1-weighted images. Finally, we also computed regional GM volume from several regions of interest (ROIs) and other RSN-derived metrics including within-network functional connectivity (WNFC) and similarity measures for test-retest reliability assessment.

## Materials and Methods

### Participants

Twenty healthy volunteers (male/female = 15/5) from Nagoya University were recruited for this study. The participants’ age ranged from 20 years to 25 years (mean = 22 years, standard deviation (SD) = 1.26 years). All participants had no history of psychiatric or neurological disorder. Participants were scanned four times, twice before (sessions S1 and S2) and twice after (sessions S3 and S4) the MR gradient coil was replaced, using the same scanning protocol. The average inter-scan interval was 67.8 days between S1 and S2, 44.0 days between S2 and S3, and 31.0 days between S3 and S4. Gradient coil noise affected some of the scans in S1 and S2 (pre-replacement) but not scans in S3 and S4 (post-replacement). The study was approved by the Ethical Committee of Nagoya University Graduate School of Medicine with approval number 1014-2. All participants signed a written informed consent before joining the study.

### Magnetic Resonance Imaging

Magnetic resonance images were acquired using a Siemen’s Magnetom Verio (Siemens, Erlanger, Germany) 3.0T MRI scanner with a 32-channel head coil. For each scanning session, a T1-weighted MR image was acquired using a 3D Magnetization Prepared Rapid Acquisition Gradient Echo (MPRAGE; Siemens; Mugler and Brookeman, 1990) pulse sequence with the following imaging parameters: repetition time (TR)/MPRAGE repetition time = 7.4/2500 ms, echo time (TE) = 2.48 ms, inversion time (TI) = 900 ms, flip angle (FA) = 8 degrees, 192 sagittal slices with a distance factor of 50% and 1-mm thickness, FOV = 256 mm, acquisition matrix dimension = 256 × 256, and in-plane voxel resolution of 1.0 × 1.0 mm^2^, with a total scan time of 5 min 49 s. Resting state functional MRI scans were also acquired using gradient echo (GE) echo planar imaging (EPI) with the following parameters: TR = 2.5 s, TE = 30 ms, 39 transverse slices with a 0.5-mm inter-slice interval and 3-mm thickness, FOV = 192 mm, matrix dimension is 64 × 64, FA = 80 degrees, 3 × 3 × 3 mm^3^ voxel resolution and 198 volumes. Participants were instructed to close their eyes during the scan but not to fall asleep. Other MRI scans were also acquired during the same imaging session, however the analysis of these datasets will be reported elsewhere.

### Preprocessing for T1-Weighted MR Images

All images were preprocessed using SPM12 (Wellcome Trust Center for Neuroimaging, London, UK) running on Matlab R2016b (MathWorks, Natick, MA, USA). The T1-weighted images were first segmented into component images including GM, white matter (WM), cerebrospinal fluid (CSF), and other non-brain tissues using SPM12’s segmentation approach. A common template for the GM images was created using DARTEL (Diffeomorphic Anatomical Registration using Exponentiated Lie algebra; Ashburner, 2007), which was then normalized to the Montreal Neuroimaging Institute (MNI) standard space. The obtained transformation information, together with the deformation fields from DARTEL, were used to normalize the component images to MNI. The normalized images were modulated to preserve the amount of signal from each region, re-sampled to an isotropic voxel size equal to 2 × 2 × 2 mm^3^, and smoothed using an 8-mm full-width-at-half-maximum (FWHM) Gaussian filter. These preprocessed images were then used in the succeeding analysis.

### Voxel-Based Morphometry

To compare differences between sessions at the voxel level, we used VBM, a commonly used approach for the analysis of T1-weighted images. For this, the preprocessed GM images were entered into a paired sample *t*-test to identify changes between sessions using SPM12. In particular, we performed S1 vs. S2 and S3 vs. S4 to compare datasets obtained using the same gradient coil as well as S1 vs. S3, S1 vs. S4, S2 vs. S3, and S2 vs. S4 to compare datasets obtained from different gradient coils. Resulting statistical maps were corrected for multiple comparisons using a family-wise error (FWE) correction rate with *p* < 0.05.

### Reliability Assessment of Regional GM Volume

Aside from voxel-wise comparisons to assess differences between sessions in anatomical (T1) images, we also estimated other metrics for test-retest reliability assessment. These metrics were then entered into an intra-class correlation (ICC) analysis, where ICC is defined as:

$$ICC(3,1)=\frac{BMS-EMS}{BMS+(k-1)EMS}$$

In the above equation, *BMS* is the between-subject variance, *EMS* is the condition error variance, and *k* is the number of conditions (Shrout and Fleiss, 1979). ICC values range from 0 to 1, with higher values (i.e., closer to 1) indicating that the between-subject error dominates, while lower values (i.e., closer to 0) indicating that the effect of conditions dominates the error.

For this analysis, we used regional GM volumes computed from the preprocessed GM images from several ROIs defined by the AAL template (Tzourio-Mazoyer et al., 2002), which divides the whole GM into 116 ROIs. The estimated regional GM volume for each ROI from all participants and sessions were then entered into the ICC analysis.

### Preprocessing for Resting State fMRI Data

For the resting state fMRI data, the first five volumes were discarded to account for the initial image inhomogeneity. The remaining images were then slice-time corrected relative to the middle slice, realigned to the mean functional image, co-registered to the bias-corrected anatomical image, normalized to MNI space using the transformation information obtained from the segmentation of the individual anatomical image, resampled to an isotropic voxel size equal to 2 × 2 × 2 mm^3^, and finally smoothed using an 8-mm FWHM Gaussian blurring kernel. To correct for head motion, the six estimated realignment parameters (three for translation and three for rotation), the parameters’ square, difference, and difference square were regressed out from the preprocessed data (Power et al., 2014). Mean signals from selected ROIs within WM and CSF and the global signal, plus the temporal difference of these signals, were also removed. The cleaned data were then bandpass filtered within 0.01–0.1 Hz.

### Group Independent Component Analysis

Group ICA was performed using Multivariate Exploratory Linear Optimized Decomposition into Independent Components (MELODIC; Beckmann et al., 2005), a component of the FSL software package^1^ to extract group-level RSNs. We used a temporal concatenation approach in MELODIC by temporally concatenating all preprocessed resting state fMRI data from all participants in all sessions. Using data from all participants and sessions in one group ICA maximizes sensitivity and avoids the problem of identifying similar components from different sessions if ICA was performed per session. We ext­racted 30 group-level independent components (ICs) to be consistent with the way the RSN templates^2^ we used were generated (Shirer et al., 2012). These group ICs were then used in the succeeding dual regression analyses (Filippini et al., 2009).

### Dual Regression Analyses

To extract participant-specific RSNs for each session, we used dual regression analysis. For each preprocessed resting state fMRI data, the identified full set of group ICs obtained from group ICA were used as spatial regressors and regression parameters were estimated at each time point giving a series of parameter estimates associated with each group IC. The estimated time courses of the regression parameters for all group ICs were then used as temporal regressors in the second regression analysis using the same resting state fMRI data to construct participant- and session-specific RSNs associated with the group RSNs. These participant-specific RSNs were then used in paired sample *t*-tests using nonparametric permutation testing (Nichols and Holmes, 2002) with 5000 permutations to identify regions that showed significant differences in functional connectivity. We compared RSNs generated using datasets obtained from the same gradient coil (S1 vs. S2 and S3 vs. S4) and RSNs generated using datasets from different gradient coils (S1 vs. S3, S1 vs. S4, S2 vs. S3, and S2 vs. S4). For all analyses, a threshold-free cluster enhancement technique (Smith and Nichols, 2009) was used and the resulting statistical maps were corrected for multiple comparisons by controlling FWE rate with *p* < 0.05.

We performed three dual regression analyses to examine the contribution of head motion and gradient coil noise in the observed differences in functional connectivity. In the first analysis, we used the bandpass filtered and cleaned resting state fMRI data from all participants without additional correction. This analysis will be referred to as the “no-correction” analysis. In the second, volumes with frame-wise displacement (FD) value greater than 0.2 were censored (scrubbing) before dual regression analysis was carried out. This analysis will be referred to as “motion-corrected” analysis. The FD value of a given volume *i* was calculated as *FD_i_* = |Δ*x_i_*| + |Δ*y_i_*| + |Δ*z_i_*| + |Δ*α_i_*| + |Δ*β*_i_| + |Δ*γ*_i_|, where Δ*x_i_* = *x*_(*i−1*)_ − *x_i_* is the difference of the estimated realignment parameter (translation) along the *x-axis* of volumes *i* and (*i* − 1). Similarly, Δ*y_i_*, Δ*z_i_*, Δ*α_i_*, Δ*β*_i_, and Δ*γ*_i_ correspond to the other translation parameters *y_i_* and *z_i_* and rotation displacements *α_i_*, *β*_i_, and *γ*_i_, respectively (Power et al., 2012). Rotation parameters were converted to displacements along the surface of a sphere with radius equal to 50 mm, this value being the approximate mean distance from the cerebral cortex to the center of the head. In the third analysis, referred to as “noise-corrected” analysis, we censored volumes with gradient coil noise before running dual regression analysis. An algorithm to detect the gradient coil noise is outlined in “Detection of Gradient Coil Noise” section.

### Other Metrics for Reliability Assessment of RSNs

For each RSN, we also computed the mean WNFC value. To estimate WNFC, we first generated RSN-specific masks. To do this, for a given RSN, we performed a one-sample *t*-test using participant-specific RSN images from all participants in all sessions using SPM12. For example, to generate the mask for the dorsal default mode network (DMN), a one-sample *t*-test was performed using participant-specific dorsal DMN images from all participants and sessions. A threshold value equal to *p* < 0.0001, corrected for multiple comparisons using FWE correction, was then applied to the resulting statistical map and all voxels with *p*-values below the threshold were included in the mask. We used a stringent threshold value to include only voxels with very significant network connectivity. To get the value of WNFC for a given RSN, the generated mask for that RSN was then applied to the participant-specific RSN image and the mean of the voxel values within the mask was computed and assigned to WNFC. The estimated WNFC values from all participants and sessions were then entered into the ICC analysis for test-retest reliability assessments.

Aside from WNFC, we also assessed the spatial similarity of the participant-specific RSNs relative to the mean RSN image computed over all participants and sessions. We used a spatial similarity measure given by *η*^2^ (Cohen et al., 2008; Choe et al., 2015):

$$\eta^{2}=1-\frac{\sum_{i=1}^{N}{\left(a_{i}-m_{i}\right)}^{2}+{\left(b_{i}-m_{i}\right)}^{2}}{\sum_{i=1}^{N}{\left(a_{i}-\bar{M}\right)}^{2}+{\left(b_{i}-\bar{M}\right)}^{2}}$$

where *a_i_* and *b_i_* are values at voxel *i* in maps *a* and *b*, respectively, *m_i_* is the mean value of the two images at voxel *i*, $\bar{M}$ is the grand mean across the mean image *m*, and *N* is the total number of voxels. Here, *a* is the participant-specific RSN and *b* is the mean RSN. *η*^2^ values can vary from 0 to 1, with 0 indicating no similarity and 1 being identical. The main advantage of this measure is that it enables the quantification of the similarity or the difference between two images instead of just the correlation between the two images.

### Detection of Gradient Coil Noise

**Figure 1:**
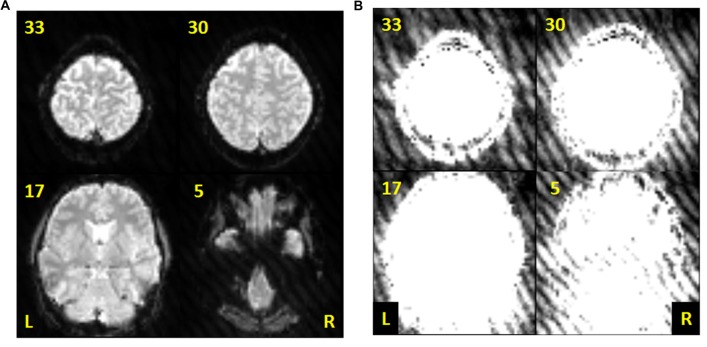
Sample echo planar images from the resting state data of subject 001 (volume 60). **(A)** Slices 5, 17, 30 and 33 displayed using intensity values ranging from 10 to 1000. **(B)** Same slices as in **(A)** but displayed using intensity values ranging from 10 to 50 to highlight the gradient coil noise throughout the image.

Since gradient coil noise could randomly appear in any volume during the scan, a method to automatically detect corrupted volumes was developed. Our data showed that the gradient coil noise has: (1) low intensity, approximately 5% to 10% of the brain’s intensity; (2) affects the entire image within a slice, both within and outside the brain; and (3) randomly appears in any volume during a given scan in all or in only some slices within the image volume. An example image is shown in Figure 1. The noise is inconspicuous when using the full intensity range to display the image (Figure 1A) and becomes apparent only when the image is displayed with intensity values ranging from 10 to 50 (Figure 1B).

To detect the gradient coil noise, we used the mean intensity of the background image outside the brain. For this, we assumed the following: (1) the mean background intensity *I_bg_* outside the brain without gradient coil noise is almost constant throughout the scan and can have different value for each slice; and (2) the mean background intensity with gradient coil noise *I_noise_* is greater than *I_bg_*. To estimate the mean background intensity, we generated an individualized outside-brain mask using the outside-brain component obtained from the segmentation of the anatomical image. This component was co-registered to the mean functional image to be in the same subject space as the realigned functional images.

**Figure 2:**
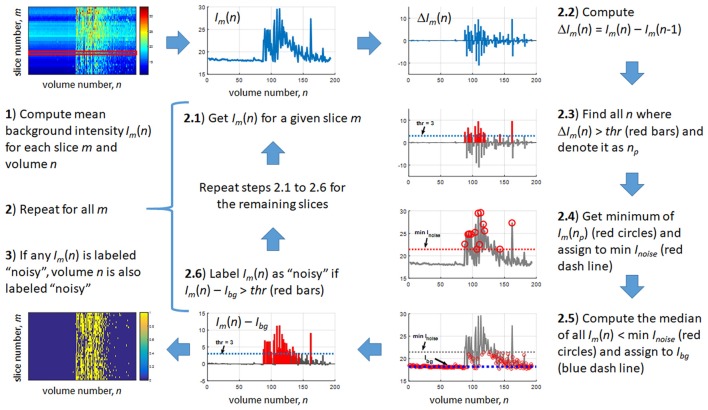
Outline of the proposed method for the automatic detection of gradient coil noise.

The detection method is outlined in Figure 2. First, the mean background intensity *I_m_*(*n*) for all slices and volumes was computed. The variables *m* and *n* are defined as slice and volume counters, respectively. An example of *I_m_*(*n*) is shown in the top left image of Figure 2. For a given slice *m* (red strip in the figure), we then extracted *I_m_*(*n*) (step 2.1) and estimated the difference in the mean intensity between successive volumes, that is, Δ*I_m_*(*n*) = *I_m_*(*n*) − *I_m_*(*n* − 1) (step 2.2). Next, we identified in step 2.3 the values of *n*, denoted as *n_p_*, where this difference was greater than a set threshold value *thr*, assumed to be the minimum jump in mean background intensity due to the gradient coil noise. This step was intended to capture sudden jumps from the background intensity *I_bg_*, although it may also be possible that this step would capture jumps from *I_noise_*. Next, we computed the minimum of *I_m_*(*n_p_*) for all *n_p_* (step 2.4) to obtain an approximation of the minimum of *I_noise_*. This min *I_noise_* value is indicated by a red dash line in the figure. To estimate *I_bg_*, we then computed the median of *I_m_*(*n*) for all *n* where *I_m_*(*n*) < min *I_noise_* (red circles) shown as a blue dash line in step 2.5. Due to the possibility that some *I_m_*(*n*) with noise would be included in the estimation of *I_bg_*, we used the median, which is more robust to outliers, instead of the mean to minimize the potential overestimation of *I_bg_*. The main purpose of steps 2.2–2.5 was to get an estimate of *I_bg_*. After obtaining this estimate, we then used it to identify volumes with noisy *m*th slice, that is, any *n* satisfying *I_m_*(*n*) > *I_bg_* + *thr*. To do this, *I_bg_* was subtracted from *I_m_*(*n*) for each *n* and any *n* where the difference was greater than *thr* was labeled as “noisy” (red bars in step 2.6). This process was then repeated for other values of *m*. Representative result of this labeling is shown in the lower left image of Figure 2 where noisy slices are shown in yellow. Volumes with at least one slice labeled as noisy were censored in the noise-corrected analyses. In order to obtain a reasonable estimate of the individualized RSNs, participants whose remaining number of volumes after noise correction was less than 120 volumes (5 min) were excluded.

## Results

### Gray Matter Changes Across Sessions

**Figure 3:**
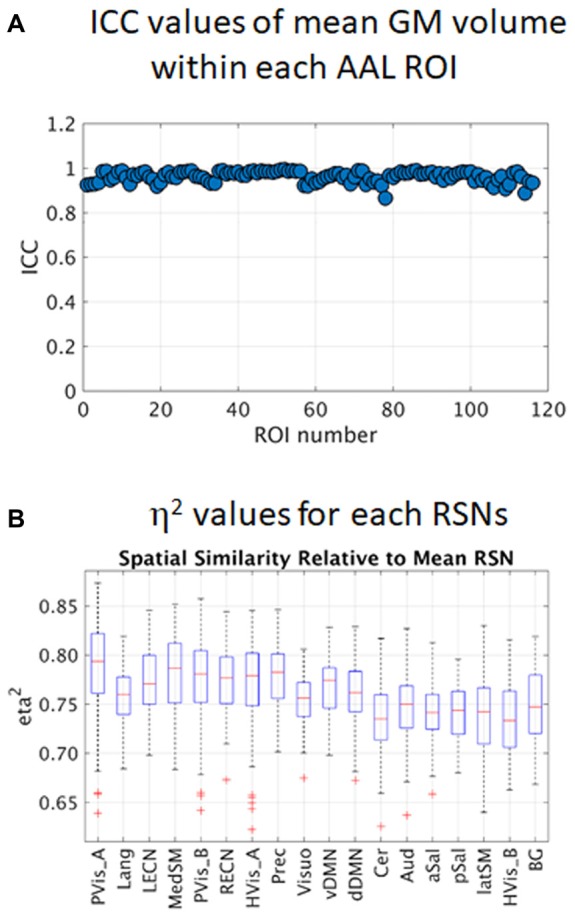
Intra-class correlation (ICC) values of regional gray matter (GM) volume within 116 AAL regions of interest (ROIs; **A**) and estimated *η*^2^ values of the 18 resting state networks (RSNs; **B**). PVis_A, primary visual (anterior); Lang, language; LECN, left executive control; MedSM, sensorimotor (medial); PVis_B, primary visual (medial); RECN, right executive control; HVis_A, high visual (medial); Prec, precuneus; Visuo, visuospatial; vDMN, ventral default mode; dDMN, dorsal default mode; Cer, cerebellum; Aud, auditory; aSal, anterior salience; pSal, posterior salience; latSM, sensorimotor (lateral); HVis_B, high visual (lateral); BG, basal ganglia.

All paired sample *t*-test comparisons of preprocessed GM images did not show any significant difference between sessions using FWE *p* < 0.05. Estimated ICC values for the 116 ROIs in the AAL template are plotted in Figure 3A. The mean ICC value across all ROIs was 0.96 (SD = 0.02) and ICC values ranged from 0.87 to 0.99. All values are close to 1 indicating that between-subject differences dominate compared to the differences among the four sessions. This result is reasonable as T1-weighted images appeared to be not affected by the faulty gradient coil.

### Low Intensity Gradient Coil Noise

Figure 1 demonstrates the effect of a faulty gradient coil on the echo planar images obtained during sessions S1 and S2. The generated gradient coil noise was not immediately visible when slices were viewed at full intensity range (Figure 1A). However, the noise, characterized by a striped pattern throughout the image, became more evident at low intensity values (Figure 1B). This gradient coil noise appeared randomly, sometimes affecting most of the volumes (and slices within the volume) in the scan, but at other times, only affecting a limited number of volumes or slices or none at all.

**Figure 4:**
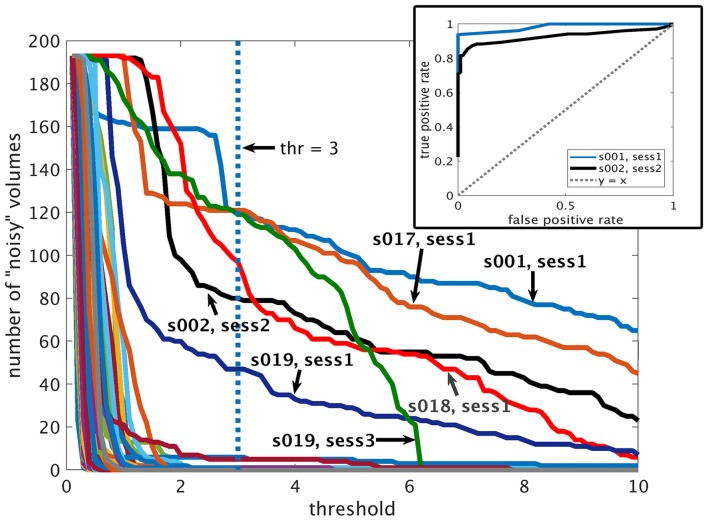
Performance of the proposed automatic detection method. Plots of the detected number of volumes with gradient coil noise as a function of the threshold value ranging from 0.1 to 10 for all datasets. Inset: receiver operating characteristic (ROC) curves for two representative datasets (from subject 001 in S1 and subject 002 in S2) affected by gradient coil noise.

**Figure 5:**
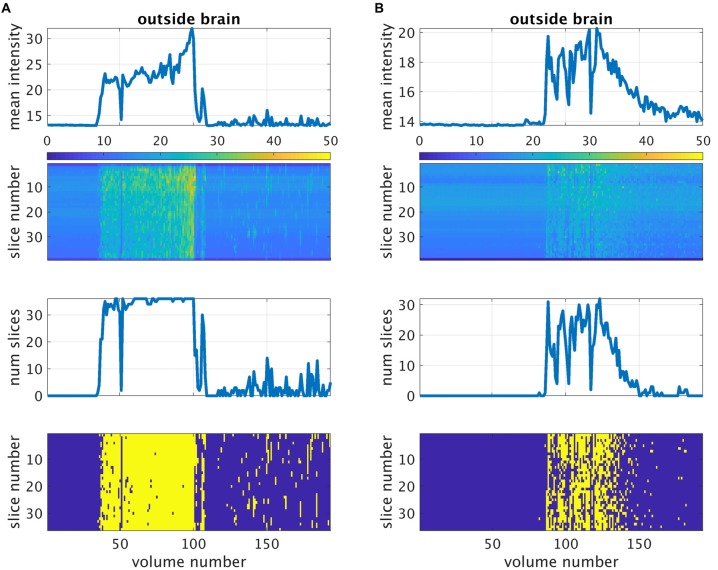
Detection of gradient coil noise using the approach described in the main text for two representative datasets from subject 001 **(A)** and subject 002 **(B)**. The first row is the mean intensity of all voxels within the individualized outside-brain mask plotted against volume number. The second row is the mean intensity per slice. The third row is the number of slices affected by the gradient coil noise plotted against volume number as detected by the described method. The last row shows the same information per slice (shown in yellow). For this analysis, the threshold value was set to 3.

The performance of the proposed method for the automatic detection of volumes with gradient coil noise is illustrated in Figure 4, which shows the number of detected “noisy” volumes as a function of the threshold. From the figure, it is evident that for clean datasets, a sharp drop in the number of detected “noisy” volumes from 193 (total number of volumes) to 0 could be observed as the threshold value approached to 2. On the other hand, datasets affected with the gradient coil noise showed a different pattern. A sharp decrease in the number of detected volumes could still be observed, but the plot’s value did not completely reach 0. Instead, the plot plateaued, then slowly decreased towards 0. We surmised that the range of threshold values where the plot plateaued could serve as the effective range of intensity values separating *I_bg_* from *I_noise_*. The receiver operating characteristic (ROC) curves constructed from two representative noisy datasets are shown in the inset. Representative output of the automatic detection method for threshold value equal to 3 indicated by the blue vertical dash line in Figure 4 is shown in Figure 5 for two resting state fMRI data severely corrupted by the gradient coil noise. Mean background intensity for the entire image (1st row) and per slice (2nd row) showed sharp intensity increases in volumes/slices corrupted by the noise. The number of slices considered as noisy for each volume is plotted in the 3rd row with the specific noisy slices shown in the last row. A value of 3 was chosen for the threshold in the succeeding analyses since most noisy datasets have plateaued at around this value.

Resting state fMRI scans severely affected by gradient coil noise included those of subject 001 in S1 (Figure 5A), subject 002 in S2 (Figure 5B), subject 017 in S1, subject 018 in S1, and subject 019 in S3. We note that the last case (subject 019 in S3) was not really due to scanner noise but rather due to one of the slices that was not properly reconstructed. Some volumes from subject 019 in S1 were also affected. Censoring volumes affected by the gradient coil noise resulted in some datasets with less than 120 volumes (5 min). For this reason, we excluded some participants in the noise-corrected dual regression analyses. The number of censored volumes due to gradient coil noise is listed in Table 1. A full list of excluded participants for different comparisons is also given in Table 2.

**Table 1:** Number of resting state functional magnetic resonance imaging (fMRI) volumes censored due to head motion (mean FD > 0.2) and gradient coil noise (threshold = 3).

Participant ID	Head motion				Gradient coil noise			
	S1	S2	S3	S4	S1	S2	S3	S4
s001	39	11	37	42	121^(*)^	0	0	0
s002	14	5	11	18	0	80^(*)^	0	0
s003	12	2	1	0	0	0	0	0
s004	17	55	55	40	0	0	0	0
s005	25	60	35	47	0	0	0	0
s006	1	4	15	3	0	0	0	0
s007	3	24	14	13	0	1	0	0
s008	50	61	30	50	0	0	0	0
s009	19	6	2	15	0	1	0	0
s010	7	7	10	55	0	0	0	0
s011	8	18	22	16	0	0	0	0
s012	6	32	74^(*)^	53	0	1	0	0
s013	11	101^(*)^	53	105^(*)^	0	6	0	0
s014	19	46	69	35	0	0	0	0
s015	29	1	31	15	0	0	0	0
s016	37	20	11	20	0	0	0	0
s017	5	2	11	39	121^(*)^	0	0	0
s018	12	11	22	3	97^(*)^	0	0	0
s019	9	2	14	4	47	0	119^(*)^	0
s020	2	3	9	11	5	0	0	0

^(*)^Indicates datasets excluded during motion-corrected or scanner-noise-corrected dual regression analyses.

**Table 2:** Participants’ data excluded in paired sample comparisons for the motion-corrected and scanner-noise-corrected dual regression analyses.

	Excluded dataset (subject ID)	
Comparisons	Gradient coil noise	Head motion
Session 1 vs. Session 2	001, 002, 017, 018	013
Session 1 vs. Session 3	001, 017, 018, 019	012
Session 1 vs. Session 4	001, 017, 018	013
Session 2 vs. Session 3	002, 019	012, 013
Session 2 vs. Session 4	002	013
Session 3 vs. Session 4	019	012, 013

*Scrubbing volumes affected by head motion and scanner noise resulted in datasets with number of volumes less than 120 (5 min)*.

### Resting State Networks

From the 30 group ICs, we identified 18 components related to known RSNs. These include primary visual (anterior and posterior), high visual (medial and lateral), language, left executive, sensorimotor (medial and lateral), right executive, precuneus, visuospatial, default mode (ventral and dorsal), cerebellum, auditory, salience (anterior and posterior) and basal ganglia networks.

### Mean Within Network Functional Connectivity

The mean and SD of WNFC values across participants for all sessions are summarized in Table 3. The computed ICC values for all RSNs are also included in the last column. Following the characterization of Li et al. (2015), 15 out of 18 RSNs had ICC values that could be considered as fair (>0.4). Three networks including salience (posterior and anterior) and lateral high visual networks had good ICC values (>0.6), while the language, precuneus, and ventral DMNs had poor ICC (<0.4). ICC values greater than 0.4 are still reasonable as fMRI results with ICC values ranging from 0.33 to 0.66 are considered as typically reliable (Bennett and Miller, 2010).

**Table 3:** Mean and standard deviation (SD) of within-network functional connectivity (WNFC) values across participants for each session and the estimated intra-class correlation (ICC) value.

Resting state networks	Mean				SD				ICC
	S1	S2	S3	S4	S1	S2	S3	S4	
Primary visual (anterior)	1.07	1.54	1.44	1.28	0.64	0.81	0.61	0.56	0.51
Language	0.58	0.61	0.63	0.58	0.18	0.16	0.14	0.15	0.36
LECN	0.59	0.57	0.57	0.56	0.18	0.15	0.12	0.14	0.56
Medial sensorimotor	0.69	0.84	0.86	0.86	0.36	0.35	0.32	0.35	0.52
Primary visual (posterior)	0.99	1.33	1.33	1.16	0.47	0.58	0.54	0.45	0.46
RECN	0.56	0.54	0.53	0.53	0.12	0.13	0.09	0.12	0.53
High visual (medial)	0.76	0.96	0.98	0.92	0.34	0.37	0.41	0.38	0.52
Precuneus	0.82	0.82	0.82	0.75	0.21	0.18	0.20	0.18	0.37
Visuospatial	0.57	0.70	0.69	0.63	0.18	0.23	0.17	0.19	0.54
Ventral DMN	0.52	0.52	0.53	0.48	0.14	0.12	0.12	0.13	0.32
Dorsal DMN	0.55	0.52	0.54	0.52	0.11	0.13	0.12	0.14	0.44
Cerebellum	0.42	0.47	0.48	0.44	0.13	0.12	0.12	0.11	0.59
Auditory	0.64	0.77	0.79	0.66	0.21	0.24	0.26	0.19	0.45
Anterior salience	0.42	0.47	0.46	0.43	0.09	0.13	0.13	0.10	0.63
Posterior salience	0.63	0.62	0.61	0.57	0.16	0.15	0.17	0.15	0.60
Lateral sensorimotor	0.72	0.82	0.82	0.77	0.29	0.26	0.23	0.27	0.57
High visual (lateral)	0.49	0.62	0.61	0.58	0.17	0.20	0.19	0.19	0.60
Basal ganglia	0.59	0.63	0.67	0.59	0.13	0.16	0.13	0.17	0.50

*LECN, left executive control network; RECN, right executive control network; DMN, default mode network; ICC, intra-class correlation*.

### Spatial Similarity of RSNs

Box plots of *η*^2^ values for the 18 RSNs are shown in Figure 3B. The degree of spatial similarity of the participant-specific RSNs compared with the mean RSN was found to be high with mean *η*^2^ values across participants and sessions ranging from 0.73 for the cerebellum (Cer) to 0.79 for the primary visual (anterior, PVis_A). Note that there were some participant-specific RSNs with *η*^2^ values that were identified as outliers (red plus sign in the plot), particularly in primary visual network (PVis_A and PVis_B) and high visual network (HVis_A), with *η*^2^ values below 0.7.

### Dual Regression Analyses

**Figure 6:**
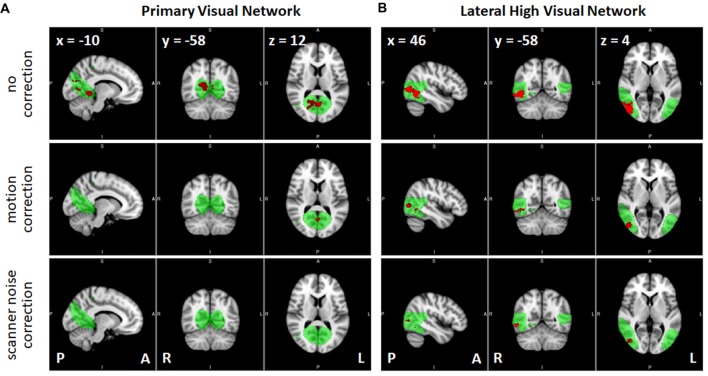
Effects of the correction methods in removing the spurious significant connectivity changes, shown in red, between sessions S1 and S2 observed in **(A)** primary visual network and **(B)** high visual network (lateral). RSNs are shown in green (group independent components (ICs) *z* > 3). P, posterior; A, anterior; L, left; R, right.

Results of the dual regression analyses are given in Tables 4–6 and Figure 6. When no correction was performed (including all participants and volumes), clusters of voxels showing significant changes in functional connectivity between S1 and S2 could be observed in several networks including primary visual, sensorimotor, high visual, visuospatial and auditory networks (Table 4). Within-network connectivity changes for the primary visual network and high visual network are shown in Figures 6A,B (1st row). We also observed significant connectivity changes in primary visual and high visual networks between S1 and S3, and in high visual network between S1 and S4. On the other hand, the cerebellum showed significant connectivity changes between S3 and S4.

**Table 4:** Voxel count for no-correction dual regression analyses.

Resting state networks	S1 vs. S2	S1 vs. S3	S1 vs. S4	S2 vs. S3	S2 vs. S4	S3 vs. S4
Primary visual^(1)^	1178	212	0	0	0	0
Language	1	0	0	0	0	0
LECN	2	0	0	0	0	0
Sensorimotor^(2)^	821	0	0	0	20	0
RECN	0	0	0	0	0	0
High visual^(3)^	1046	626	83	0	0	0
Precuneus	0	0	0	0	0	0
Visuospatial	158	2	0	0	0	0
DMN^(4)^	0	0	1	0	0	0
Cerebellum	0	0	0	3	0	56
Auditory	31	0	0	0	0	0
Salience^(5)^	0	0	0	0	0	0
Basal ganglia	0	0	8	0	16	0
Total	**3237**	**840**	**92**	**3**	**36**	**56**

*The indicated number of voxels include both increase and decrease contrasts. S1, session 1; S2, session 2; S3, session 3; S4, session 4; LECN, left executive control network; RECN, right executive control network; DMN, default mode network. NOTE: Total of ^(1)^anterior and posterior components, ^(2)^medial and lateral, ^(3)^medial and lateral, ^(4)^dorsal and ventral, and ^(5)^posterior and anterior*.

**Table 5:** Voxel count for motion-corrected dual regression analyses.

Resting state networks	S1 vs. S2	S1 vs. S3	S1 vs. S4	S2 vs. S3	S2 vs. S4	S3 vs. S4
Primary visual^(1)^	97	1	0	0	1	0
Language	3	0	0	2	0	0
LECN	26	11	0	0	2	0
Sensorimotor^(2)^	309	21	0	0	0	106
RECN	0	0	0	0	0	0
High visual^(3)^	316	258	0	0	0	17
Precuneus	0	0	0	0	0	0
Visuospatial	129	46	0	0	0	0
DMN^(4)^	0	0	0	0	0	0
Cerebellum	4	0	0	15	0	0
Auditory	0	0	0	0	0	0
Salience^(5)^	16	0	13	0	0	0
Basal ganglia	0	0	5	3	0	0
Total	**900**	**337**	**18**	**20**	**3**	**123**

*The indicated number of voxels include both increase and decrease contrasts. S1, session 1; S2, session 2; S3, session 3; S4, session 4; LECN, left executive control network; RECN, right executive control network; DMN, default mode network. NOTE: same as in Table 4*.

**Table 6:** Voxel count for gradient-coil-noise-corrected dual regression analyses.

Resting state networks	S1 vs. S2	S1 vs. S3	S1 vs. S4	S2 vs. S3	S2 vs. S4	S3 vs. S4
Primary visual^(1)^	0	0	0	0	0	0
Language	0	0	0	0	0	0
LECN	0	0	0	0	0	0
Sensorimotor^(2)^	0	0	0	1	0	0
RECN	0	0	0	0	0	0
High visual^(3)^	180	0	0	0	0	0
Precuneus	0	0	0	0	0	0
Visuospatial	0	0	0	2	0	0
DMN^(4)^	7	0	0	0	0	0
Cerebellum	0	0	0	0	0	44
Auditory	0	0	0	0	0	0
Salience^(5)^	0	0	0	0	1	0
Basal ganglia	73	0	30	33	70	0
Total	**260**	**0**	**30**	**36**	**71**	**44**

*The indicated number of voxels include both increase and decrease contrasts. S1, session 1; S2, session 2; S3, session 3; S4, session 4; LECN, left executive control network; RECN, right executive control network; DMN, default mode network. NOTE: same as in Table 4*.

Correcting for the effect of head motion by censoring volumes with FD values greater than 0.2 reduced the number of voxels exhibiting significant connectivity changes between S1 and S2 (Table 5). In particular, the number of voxels in the primary visual, sensorimotor and high visual networks was significantly reduced when head motion was taken into account. This is also evident in Figure 6 (2nd row). On the other hand, the effect of head motion correction on the connectivity changes in the visuospatial networks was relatively modest. Between S1 and S3, significant changes in connectivity in the (primary/high) visual networks was also reduced, although some other networks (LECN, sensorimotor and visuospatial) also exhibited a slight increase in the number of voxels showing significant connectivity changes. The observed changes in the cerebellum for the no-correction analysis between S3 and S4 disappeared, indicating that the observed change could be driven by motion artifact. The sensorimotor network, however, showed an increase in the number of voxels, which was not observed in the no-correction analysis. Overall, we could observe a reduction in the number of voxel showing significant connectivity changes when head motion was taken into account. The list of the number of censored volumes for each participant and session is given in Table 1 and the list of participants excluded in the motion-corrected analyses due to excessive head motion is given in Table 2.

When datasets were corrected for gradient coil noise, the number of voxels exhibiting connectivity changes was significantly reduced especially in comparisons involving S1 (Table 6). Specifically, the total number of significant voxels was reduced from 3237, 840 and 92 in no-correction analyses to 260, 0 and 30 in noise-corrected analyses in S1 vs. S2, S1 vs. S3 and S1 vs. S4, respectively. Figures 6A,B (3rd row) shows the case for the visual networks. This clearly suggests that the observed connectivity changes were spurious and mainly due to the gradient coil noise in resting state fMRI data acquired before the gradient coil was replaced. The remaining voxels with significant connectivity changes could be due to other factors including motion artifact. For instance, the number of voxels in high visual network was reduced when motion was taken into account. Thus, the remaining 180 voxels in this network could just be due to head motion. In addition, the 44 voxels observed in the cerebellum in S3 vs. S4 comparison could also be due to motion as this cluster disappeared when the analysis was corrected for motion. Compared to the no-correction analyses, we also observed an increase in the number of voxels showing significant connectivity changes in the basal ganglia network especially in comparisons involving S2. Again, this could be driven by head motion as the number of voxels with significant connectivity changes in this network were very limited in the motion-corrected analyses (Table 5) and subject 013 had high motion data in both S2 and S4 (Table 1). Based on these results, censoring volumes affected by gradient coil noise could lead to reproducible RSNs on datasets obtained with the same gradient coil (i.e., S1 vs. S2 and S3 vs. S4) and between different gradient coils (i.e., S1 vs. S3, S1 vs. S4, S2 vs. S3 and S2 vs. S4).

Unfortunately, simultaneously censoring both gradient coil noise and head motion was not possible since it would result to several datasets with volumes less than 120. However for completeness, we included in Supplementary Table S1 (excluded data sets) and Supplementary Table S2 (voxel count) the case where both gradient coil noise and head motion were simultaneously censored lowering the minimum number of volumes to 96 (4 min) and for a limited data set.

## Discussion

We examined the effect of low intensity gradient coil noise and gradient coil replacement on the reproducibility of T1-weighted images and resting state fMRI data. Our main findings showed that: (1) T1-weighted images were not affected by this noise and metrics derived from T1 were highly reproducible even after gradient coil replacement; (2) in spite of being low in intensity, the observed gradient-coil-generated noise could introduce significant connectivity changes in several RSNs affecting WNFC values, which consequently, resulted to only fair ICC values; and (3) these spurious connectivity changes could be eliminated by censoring volumes corrupted by gradient coil noise in the same manner as minimizing the effects of head motion by censoring volumes with high motion (Power et al., 2015). After gradient coil noise correction, spurious connectivity changes were significantly reduced, if not completely removed, in comparisons between sessions within the same gradient coil (S1 vs. S2) and across different gradient coils (e.g., S1 vs. S3 and S2 vs. S4) suggesting the reproducibility of RSNs before and after gradient coil replacement.

### RSN Reproducibility

Previous studies have shown that RSNs are reproducible within participants over durations of weeks to months (Damoiseaux et al., 2006; Wisner et al., 2013) and even longer periods (Guo et al., 2012; Choe et al., 2015). A study comparing test-retest reliability of resting state fMRI data using temporal signal to noise ratio (tSNR) within two RSNs as a metric after a major hardware repair had also shown consistent tSNR across conditions (Huang et al., 2012). However, the authors did not mention the presence of noise in the used resting state fMRI data before gradient coil replacement or considered the effect of noise on the reproducibility of resting state fMRI.

In this study, we have focused on the effect of gradient coil noise on the reproducibility of RSNs. Our results showed that before the gradient coil was changed, the gradient coil noise significantly affected several RSNs in terms of spurious detectable connectivity changes. The gradient coil noise also affected the estimates of the mean WNFC as can be seen in Table 3, where the primary visual (anterior) and high visual (medial) networks have relatively lower WNFC values in S1, where most of the gradient coil noise was observed, compared to other sessions. This, in turn, could have affected the estimates of the ICC values resulting in a relatively fair reliability of the networks. The box plots of the spatial similarity measure *η*^2^ (Figure 3B) showed several outliers which could again be driven by the presence of the gradient coil noise in some resting state fMRI data before the gradient coil was replaced.

To mitigate this issue, we introduced a technique which significantly reduced, if not completely removed, spurious connectivity changes driven by the gradient coil noise in some resting state fMRI data. We employed a similar method, called scrubbing or censoring, used to minimize motion artifacts in the presence of large head motion (Power et al., 2015). Application of this method reduced the number of voxels exhibiting spurious connectivity changes in all networks, except in high visual and basal ganglia networks, in comparisons involving S1. The remaining voxels could be just due to other factors including motion artifact. The disadvantage of the method is that it reduced the number of volumes included in the analysis and led to the exclusion of the entire dataset for extremely noisy scans. In this case, a method that would only remove the noise from the image without discarding the volumes in the analysis would be more beneficial.

Another concern with censoring relates to the differences in the number of volumes included in the analysis (degrees of freedom) for each participant, which could introduce variability in the estimates of the correlation values. In our analysis, we set the minimum number of volumes to 120 (5 min) and datasets with volumes less than this minimum were excluded. As shown by Van Dijk et al. (2010), estimates of correlation strengths started to stabilize with acquisition times as brief as 5 min. We can therefore assume that in the preceding analyses the variability introduced due to the differences in the number of volumes would be minimal. Moreover, for the noise-corrected analyses, most of the datasets with censored volumes were excluded and only one dataset (subject 019 in S1) where the number of censored volumes was greater than six was included. The findings of these analyses were therefore minimally affected by this issue. For the motion-corrected analyses, the number of censored volumes for each participant did vary from session to session. Thus, the results of these analyses should be carefully interpreted with this potential limitation.

### T1 Anatomical Image

With ICC values close to 1, our ROI-based analysis of GM images revealed high reproducibility across gradient coil replacement. This is further confirmed using a direct voxel-wise comparison between sessions showing no significant changes observed in statistical maps using a statistical threshold of *p* < 0.05 FWE-corrected for multiple comparisons, a standard practice for voxel-based morphometric analysis. This is consistent with the observation that the faulty gradient coil had not yet affected T1-weighted images and had not introduced any artifact into these images. This finding is also consistent with that of Huang et al. (2012) showing high reproducibility of the volumes of selected ROIs as stability metrics for T1 images after gradient coil replacement.

### Detection of Gradient Noise

We also proposed a reliable method to automatically detect the gradient coil noise from the resting state fMRI data. The method relied on the noise characteristics observed in our dataset. In particular, it assumed that the mean intensity of the background image (outside brain) was constant in the absence of gradient coil noise. Under this condition, the method worked quite satisfactorily. It may also work with other types of randomly occurring image artifacts such as image reconstruction failures as demonstrated in one of the resting state fMRI data. However, its general applicability to other types of noise will depend on the characteristics of the noise being considered.

Another important issue to consider for the method’s general applicability is the proper choice of the threshold value to use in detecting noisy slices or volumes. Here, we used a threshold value of 3, which appeared to be optimal for the current datasets. However, this may not be the case for other datasets. Choosing the appropriate threshold value is important especially when using censoring to minimize spurious findings. Interestingly, the plots showing the number of detected “noisy” volumes as a function of the threshold (Figure 4) could provide some useful hints in selecting the appropriate threshold value. As we have mentioned in the results section, the observed plateau in the plot could indicate the range of threshold values separating *I_bg_* from *I_noise_*. Given this, the value where the plot starts to plateau could therefore be used as the appropriate threshold. This worked for most of our noisy datasets, although its general applicability still remains to be validated.

## Conclusion

In summary, hardware-related noise such as those due to a faulty gradient coil could significantly affect the reproducibility of RSNs. In spite of its low intensity, this type of noise could introduce spurious connectivity changes in several RSNs, which could be easily mistaken as valid changes. By censoring corrupted image volumes during the analysis, the number of spurious connectivity could be significantly minimized if not completely removed. Applying this correction method could also make RSNs reproducible even after gradient coil replacement. These findings suggest that, with an appropriate correction method, resting state fMRI datasets affected by hardware problems could still be used to generate consistent and reproducible RSNs.

## Author Contributions

EB, SK, HI, HW, SM, SN and GS conceived and designed the study. EB, ET, YY, YO, MK, TO, SK and HI performed the experiments and analyzed the dataset. EB, HI, HW and SM wrote the draft of the manuscript and all authors reviewed and approved the final manuscript.

## Conflict of Interest Statement

The authors declare that the research was conducted in the absence of any commercial or financial relationships that could be construed as a potential conflict of interest.

## Footnotes

^1^http://fsl.fmrib.ox.ac.uk/fsl/fslwiki/

^2^http://findlab.stanford.edu/functional_ROIs.html
