# Supplementary material for: Effects of Gradient Coil Noise and Gradient Coil Replacement on the Reproducibility of Resting State Networks
Source: Front Hum Neurosci. 2018 Apr 19;12:148. doi: 10.3389/fnhum.2018.00148 (PMC5917444; doi:10.3389/fnhum.2018.00148)
Supplement: Supplementary file 1 [file Table_1.docx]

**Supplemental Table S1**. Participants’ data excluded in paired sample comparisons for the scanner noise and motion corrected analysis.

| Comparisons | Excluded Data (Subject ID) |
| --- | --- |
| Session 1 vs Session 2 | 001, 013, 017, 018 |
| Session 1 vs Session 3 | 001, 017, 018, 019 |
| Session 1 vs Session 4 | 001, 013, 017, 018 |
| Session 2 vs Session 3 | 013, 019 |
| Session 2 vs Session 4 | 013 |
| Session 3 vs Session 4 | 013, 019 |

Scrubbing volumes affected by scanner noise and motion resulted in datasets with number of volumes that is less than 96 (4 min).
