# Supplementary material for: Effects of Gradient Coil Noise and Gradient Coil Replacement on the Reproducibility of Resting State Networks
Source: Front Hum Neurosci. 2018 Apr 19;12:148. doi: 10.3389/fnhum.2018.00148 (PMC5917444; doi:10.3389/fnhum.2018.00148)
Supplement: Supplementary file 2 [file Table_2.docx]

**Supplemental Table S2**. Voxel count for scanner noise and motion corrected analysis lowering the minimum number of volumes to 96 (4 min) and using limited number of data sets (see Supplemental Table S1).

| Resting State Networks | S1vsS2 | S1vsS3 | S1vsS4 | S2vsS3 | S2vsS4 | S3vsS4 |
| --- | --- | --- | --- | --- | --- | --- |
| Primary Visual^1)^ | 1 | 0 | 0 | 0 | 1 | 0 |
| Language | 0 | 0 | 0 | 0 | 0 | 0 |
| LECN | 2 | 0 | 0 | 0 | 0 | 0 |
| Sensorimotor^2)^ | 0 | 0 | 0 | 0 | 0 | 21 |
| RECN | 8 | 0 | 0 | 0 | 0 | 0 |
| High Visual^3)^ | 57 | 5 | 0 | 9 | 0 | 0 |
| Precuneus | 0 | 0 | 0 | 0 | 0 | 0 |
| Visuospatial | 86 | 0 | 0 | 0 | 0 | 0 |
| DMN^4)^ | 33 | 0 | 0 | 0 | 0 | 0 |
| Cerebellum | 0 | 0 | 0 | 0 | 0 | 11 |
| Auditory | 0 | 0 | 0 | 0 | 4 | 1 |
| Salience^5)^ | 0 | 0 | 3 | 0 | 0 | 1 |
| Basal Ganglia | 13 | 0 | 6 | 0 | 14 | 0 |
| Total | **200** | **5** | **9** | **9** | **19** | **34** |

The indicated number of voxels include both increase and decrease contrasts. S1, session 1; S2, session 2; S3, session 3; S4, session 4; LECN, left executive control network; RECN, right executive control network; DMN, default mode network. NOTE: Total of ^(1)^ anterior and posterior components, ^(2)^ medial and lateral, ^(3)^ medial and lateral, ^(4)^ dorsal and ventral, and ^(5)^ posterior and anterior.
